# Multivariate spatio-temporal modelling for assessing Antarctica's present-day contribution to sea-level rise

**DOI:** 10.1002/env.2323

**Published:** 2015-01-16

**Authors:** Andrew Zammit-Mangion, Jonathan Rougier, Nana Schön, Finn Lindgren, Jonathan Bamber

**Affiliations:** a School of Geographical Sciences, University of BristolBristol, BS8 1SS, U.K.; b Department of Mathematics, University of BristolBristol, BS8 1TW, U.K.; c Department of Mathematical Sciences, University of BathBath, BA2 7AQ, U.K.

**Keywords:** Multivariate modelling, parallel MCMC, sea-level rise, spatio-temporal statistics, stochastic partial differential equations

## Abstract

Antarctica is the world's largest fresh-water reservoir, with the potential to raise sea levels by about 60 m. An ice sheet contributes to sea-level rise (SLR) when its rate of ice discharge and/or surface melting exceeds accumulation through snowfall. Constraining the contribution of the ice sheets to present-day SLR is vital both for coastal development and planning, and climate projections. Information on various ice sheet processes is available from several remote sensing data sets, as well as *in situ* data such as global positioning system data. These data have differing coverage, spatial support, temporal sampling and sensing characteristics, and thus, it is advantageous to combine them all in a single framework for estimation of the SLR contribution and the assessment of processes controlling mass exchange with the ocean.

In this paper, we predict the rate of height change due to salient geophysical processes in Antarctica and use these to provide estimates of SLR contribution with associated uncertainties. We employ a multivariate spatio-temporal model, approximated as a Gaussian Markov random field, to take advantage of differing spatio-temporal properties of the processes to separate the causes of the observed change. The process parameters are estimated from geophysical models, while the remaining parameters are estimated using a Markov chain Monte Carlo scheme, designed to operate in a high-performance computing environment across multiple nodes. We validate our methods against a separate data set and compare the results to those from studies that invariably employ numerical model outputs directly. We conclude that it is possible, and insightful, to assess Antarctica's contribution without explicit use of numerical models. Further, the results obtained here can be used to test the geophysical numerical models for which *in situ* data are hard to obtain. © 2015 The Authors. *Environmetrics* published by John Wiley & Sons Ltd.

## 1. Introduction

Antarctica is the largest fresh-water resource on Earth and, potentially, the largest contributor to global sea-level rise (SLR). Its ice sheet is widely believed to be losing on the order of 50 Gt year^−1^ (Shepherd *et al.*, 2012; Gunter *et al.*, 2014; Rignot *et al.*, 2008), and this rate is likely to increase (Rignot *et al.*, 2011b). SLR has global implications and accurate assessment of present-day rates, sources and their uncertainties are essential for prediction, and hence the development and implementation of effective urban, eco-preservation and flood defence strategies (Vaughan *et al.*, 2013).

A review of the difficulties associated with estimating the SLR contribution from Antarctica and the assumptions of current approaches that tackle this problem is given in Zammit-Mangion *et al.* (2014). The primary difficulty is that of under-determination (lack of mixture diversity), because the number of observed linear combinations (using satellite and global positioning system (GPS) instruments) is less than the number of processes influencing the instruments. Zammit-Mangion *et al.* (2014) showed that under-determination, for this problem, can be tackled using a hierarchical modelling framework (Cressie & Wikle, 2011). Their approach, which as a proof of concept focused on only a part of the Antarctic ice sheet, took advantage of differing spectral characteristics to improve the separation of the individual processes from the data. The analysis was time invariant and considered the mean rate of height change of each process, between 2003 and 2009, at every spatial location, to be the variable of interest.

In this article, we extend Zammit-Mangion *et al.* (2014) in several ways. (i) We consider *spatio-temporal* changes in height change instead of just spatial rates. This has two benefits, first differing temporal characteristics across the processes play a key role in improving the predictive performance on the marginal processes (see Cressie *et al*. (2010) Section 4) and second, considering temporal characteristics helps in source separation, that is determining which processes are causing the observed change. (ii) We extend the assessment to the whole of Antarctica and also consider an extra (fifth) process layer to explain fine-scale variation that is clearly affecting one of the data sets: satellite altimeter measurements of height change. (iii) To cater for the computational demands, which emerge at these scales and data volumes, we design an inference scheme that is parallelisable and demonstrate the potential speed-ups on a high-performance computer. As in Zammit-Mangion *et al.* (2014), the approach is sensitive to our prior beliefs, and we need to take certain justified modelling assumptions before running update schemes. We note that spatio-temporal inversion using a process-based framework such as that proposed here has been used elsewhere in studies of the cryosphere, for example by Hurkmans *et al.* (2012), and from a physical-statistical modelling perspective, by Berliner *et al.* (2008). However, these works concentrated on a small region such as a single outlet glacier or ice stream and do not consider an under-determined problem. For reference, the area of the Antarctic ice sheets exceeds 13 M km^2^, and some processes operate over spatial scales of 20 km^2^. This work is the first of its kind to provide an ice-sheet wide spatio-temporal inversion of all dominating ice sheet processes.

Using the work in Zammit-Mangion *et al.* (2014) as a foundation, the framework is constructed with standard hierarchical spatio-temporal modelling tools (Cressie & Wikle, 2011) in Section 2. In Section 3, we outline the use of numerical models to elicit informative priors and detail a parallelisable inference scheme to infer all other unknowns. In Section 4, we validate and visualise the results and compare them to other works. Section 5 concludes. Code used in this work is available in *R software* from the project website http://www.rates-antarctica.net/. A package and accompanying vignette facilitating most of the operations described in this work is available from the first author's git website https://github.com/andrewzm/MVST.

## 2. Hierarchical modelling

We will follow a hierarchical modelling approach to the problem, as exemplified in works such as Berliner *et al.* (2000) and Wikle & Berliner (2007). The interested reader is referred to Cressie & Wikle (2011) for a comprehensive review. In summary, the model we will define is structured with (i) an observation layer detailing the interactions between the instruments and the processes under investigation; (ii) the process layer containing information on the spatio-temporal nature of the processes; and (iii) the parameter layer encoding prior beliefs on unknowns, which appear in any of the first two layers.

The nature of the observations in this application are sufficiently complex to warrant a split of the top layer into two sublayers. The first layer identifies interactions that may occur between the observations themselves. This is primarily included to cater for averaging effects occurring in the observations with large spatial footprint. The second layer describes the measurement mapping between the process layer and the instrument itself. The ‘process’ we consider is a superposition of a multivariate spatio-temporal model and a fine-scale effect. Although the fine-scale effect is not of direct interest, it is clearly present in the altimetric observations and thus needs to be considered.

**Figure 1:**
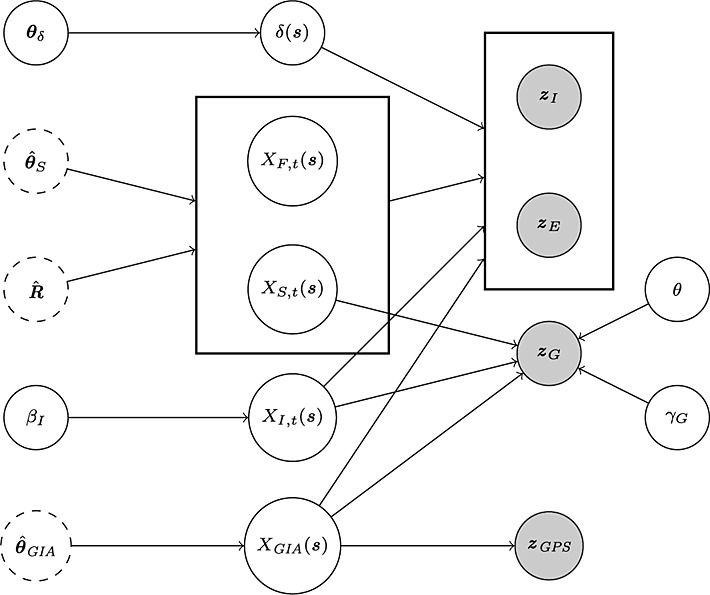
Graphical model for the problem under study. Variables on the left are parameters pertaining to the underlying spatio-temporal processes. *θ*_*δ*_ denotes the fine-scale variation (*δ*(*s*)) parameters, 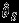, parameters relating to the surface and firn processes (*X*_*S*,*t*_(*s*) and *X*_*F*,*t*_(*s*)), 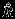 the interaction between these two processes, *β*_*I*_ the weightings of covariates associated with the model for height change due to ice dynamics (*X*_*I*,*t*_(*s*)) and 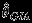 parameters appearing in the model for glacio-isostatic adjustment (*X*_*GIA*_(*s*)). Variables in the middle denote the spatio-temporal processes, while variables on the right denote the observations (filled circles). The parameters *θ* and *γ*_*G*_ are parameters appearing in the observation equation for *z*_*G*_. Rectangles denote groupings: the altimetry data sets *z*_*E*_ and *z*_*I*_ are treated identically (same observation characteristics), while *X*_*S*,*t*_(*s*) and *X*_*F*,*t*_(*s*) are, jointly, a multivariate spatio-temporal process. Dashed circles enclose the parameters estimated using maximum likelihood from data/numerical models prior to computing the Bayesian update. The pseudo observations are omitted for clarity

In this work, we will adopt a hybrid approach where we take advantage of the geophysical numerical models to estimate some of the parameters and infer other parameters from data using a Markov chain Monte Carlo (MCMC) scheme. The fourth layer describes prior distributions over these latter parameters, which appear both in the first observation layer and in the component describing the fine-scale variation. A graphical model showing all parameters (both estimated and random), processes and data sets is given in Figure 1 for use as reference throughout this section.

### 2.1. Data preprocessing and the data model

There are, broadly, two satellite-based techniques used when assessing the SLR contribution of the Antarctic ice sheet. The first, satellite altimetry, is a technique for measuring ice-sheet height change, while the second, gravimetry, is a technique for measuring changes in the Earth's gravity field, the geoid, which can be used to infer mass changes. While the former produces point-referenced data, the latter is a potential field that generally produces large spatial footprints (often expressed as a spherical harmonic expansion). In this work, we made use of the most informative data sets available to date, from the

1. laser-based Ice Cloud and Elevation Satellite (ICESat, altimetry),
2. radar-based Environment Satellite (EnviSat, altimetry),
3. Gravity Recovery and Climate Experiment (GRACE, gravimetry).

In addition to these, we also use GPS data, which provide a direct reading of bedrock uplift rates.

**Figure 2:**
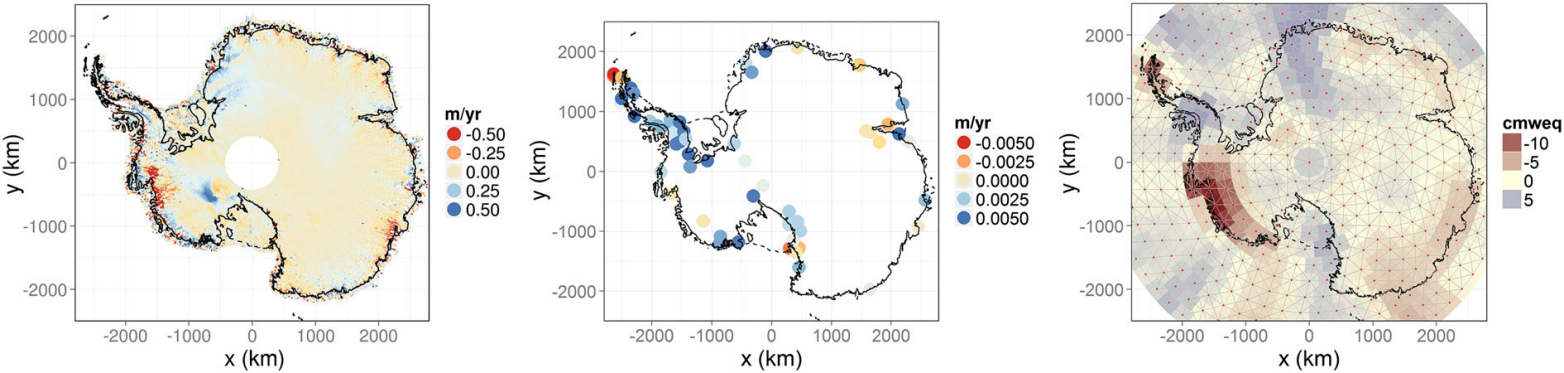
Snapshots from some of the data sets employed in this study. Left: Ice Cloud and Elevation Satellite elevation rates in 2006, Centre: global positioning system elevation rates in 2006 and Right: Gravity Recovery and Climate Experiment mass anomalies in 2006 in centimetres water equivalent (cmweq) with overlayed neighbourhood graph. The continent's grounding line and coastline are denoted using solid and dashed lines, respectively

The unit of interest is *height change* in m year^−1^ due to each of the four processes of interest. We therefore preprocessed the data accordingly to obtain spatially distributed height or mass (in case of GRACE) rates and associated errors for each year. The altimetry data were processed on a 20×20 km grid as in Riva *et al.* (2009), while the GRACE observations were preprocessed as in Zammit-Mangion *et al.* (2014). The time interval for this study was restricted to the overlapping epochs of the operating instruments, namely 2003–2009. GRACE data for 2003 were, however, omitted on recommendation of Luthcke (personal communication) because of issues with data quality for this year in the product we were using. We also included pseudo point-referenced observations to weakly constrain the height rates due to ice, surface and firn processes to zero outisde the continent's coast. These observations, of which we have just over 24 000, were placed on the triangulation vertices of the relevant processes (Section 2.4) in the ocean and were assigned a variance of 0.001. In all, we make use of just over 365 000 observations, 2832 of which have a large spatial footprint (corresponding to the GRACE data). Snapshots from ICESat, GPS and GRACE in 2006 are shown in Figure 2, and the role of the data sets used (excluding the pseudo-data) is summarised in Table 1.

**Table 1:** Observation data sets employed, together with type of spatial footprint, the processes which they are able to detect changes in and the number of values available for the temporal horizon under consideration (2003–2009, GRACE 2004–2009)

Data	Footprint	Detects	Number	Symbol
ICESat	point	fine scale, surface, firn, ice processes and GIA	193 914	***z***_*I*_
EnviSat	point	fine scale, surface, firn, ice processes and GIA	164 551	***z***_*E*_
GRACE	polar polygons (≈ 40 000 km^2^)	surface, ice processes and GIA	2832	***z***_*G*_
GPS	point	GIA	147	***z***_*GPS*_

ICESat, Ice Cloud and Elevation Satellite; EnviSat, Environment Satellite; GRACE, Gravity Recovery and Climate Experiment; GPS, global positioning system; GIA, glacio-isostatic adjustment.

#### 2.1.1. First data layer

We use the first data layer to describe the mapping between the observations and the processes. Denote the ICESat rates at year *t* as ***z***_*I*,*t*_, the EnviSat rates as ***z***_*E*,*t*_, the GRACE rates as ***z***_*G*,*t*_, the GPS rates as ***z***_*GPS*,*t*_ and let 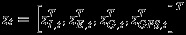. Further, denote the respective errors as 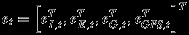. Then, we can write the first data layer as


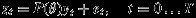
(1)

where 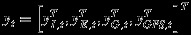 is defined in Section 2.1.2 and ***P***(*θ*) = bdiag(***I***_*I*_,***I***_*E*_,***P***_*G*_(*θ*),***I***_*GPS*_), where bdiag(·) places its arguments along a block diagonal. We assume that the altimetry and GPS observations are point-referenced independent records of the first layer, hence the use of the identity matrix ***I***_*i*_. On the other hand, it is well known that the effective resolution of GRACE is less than that supplied (Luthcke *et al.*, 2013), and the role of ***P***_*G*_(*θ*) is to cater for this observation interaction. ***P***_*G*_(*θ*) is thus defined as


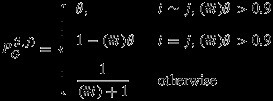
(2)

where (*#i*) denotes the number of neighbours of observation *i* and ∼ denotes ‘neighbour of’. Equation (2) describes the proportion of signal (*#i*)*θ* that should be attributed to the spatial regions associated with the neighbouring observations. If (*#i*)*θ* exceeds 0.9 (indicative of poor localisation), the observation is assumed to be an equal average of itself and its neighbours. Two GRACE observations are marked as neighbours if their geometric centres are distanced by less than 450 km, see Figure 2 (right).

For the GRACE signal, preprocessing techniques might also induce an under-estimation on the variance of ***e***_*G*,*t*_ (Zammit-Mangion *et al.*, 2014). To allow for this, we redefine 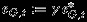 where 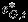 is the original supplied error and *γ*_*G*_ is an inflation parameter, which, together with *θ* (the strength of neighbourhood interaction), needs to be estimated.

#### 2.1.2. Second data layer

The second data layer models the relationship between the processes, fine-scale variation *δ*_*t*_(***s***) and the data. There are four dominating physical processes that need to be considered when estimating SLR contribution. (i) The first is a solid-Earth process known as glacio-isostatic adjustment (GIA), the response of the lithosphere to past changes in ice loading that took place, primarily, at the end of the last glacial, around 12 KYr ago; (ii) on top of the bedrock sits the ice, which constantly flows from the interior to the ocean; (iii) between the surface snow and the ice at depth lies a firn layer that has a density between that of snow and ice and that is constantly compacting; (iv) at the surface, the mass balance is affected mostly by changes in precipitation, sublimation and (limited) melt-water run-off. The role of these layers and the way they interact with the instrumentation are discussed in greater detail in Zammit-Mangion *et al.* (2014). In the current geophysical setting, the fine-scale variation is due to localised (fine-scale) precipitation anomalies (Isaksson & Karlen, 1994), which are most evident in areas of rapidly varying topography (Richardson *et al.*, 1997).

The model for the second layer is as follows


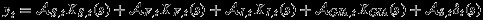
(3)

where the {*X*_*i*,*t*_} denote the change in height (in m year^−1^) due to processes that contain some spatio-temporal structure (as opposed to *δ*_*t*_); the overall surface process anomaly (*S*), firn densification (*F*), ice dynamics/subshelf melting (*I*) and GIA (*GIA*), respectively. Note that because we are operating in a multivariate setting, we have not modelled fine-scale variation to be part of a particular process of interest (e.g. Katzfuss & Cressie, 2011 and Kang & Cressie, 2011), so that fine-scale effects from all processes, if present, are captured in *δ*_*t*_(***s***).

Each element in the vectors of linear operators 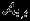 maps the physical process *X*_*i*,*t*_(***s***) or the fine-scale variation into the observation space. Each element in 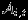 depends on the type and the location of the observation. For the data sets described earlier, it is sufficient to let each element in 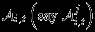 to be an integral operator of the form


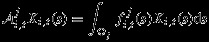
(4)

for *i*∈{*S*,*F*,*I*,*GIA*,*δ*}, where Ω_*j*_ is the observation footprint, 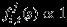 or 1/|Ω_*j*_| in the case of a purely accumulating or averaging observation, respectively, and 
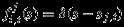
 in the case of a point-referenced observation at location ***s***_*j*_ at time *t*. For the altimetry data sets, pseudo-data sets and GPS, because their spatial support is relatively small, we assumed point-referenced observations and thus let Ω_*j*_=Ω (the entire domain) and 
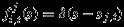
 for all processes. For the GPS and pseudo observations, we let 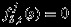 for each *j* because these observations are not affected by the fine-scale process.

For GRACE, we let 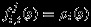 for each *i*∈{*I*,*S*,*F*,*GIA*,*δ*}, where each *ρ*_*i*_(***s***) is a spatially varying density map used to convert the height change to the detected mass change. *ρ*_*I*_(***s***):=*ρ*_*I*_ is the density of ice (917 kg m^−3^), *ρ*_*S*_(***s***) is the density at which surface process fluctuations occur (spatially varying between 300 and 600 kg m^−3^) obtained from the Regional Atmospheric Climate Model (RACMO, Lenaerts *et al.*, 2012), *ρ*_*F*_(***s***):=*ρ*_*F*_=0 because firn compaction is a mass-preserving process and *ρ*_*GIA*_(***s***):=*ρ*_*GIA*_ is the effective mantle density (3800 kg m^−3^) (RACMO, used to set *ρ*_*S*_(***s***), is a regional climate model that reconstructs, at a resolution of 27 km, various surface processes from global climate reanalysis data. It will also be used in Section 3 to provide information on the spatial-temporal chatacteristics of the surface processes). For GRACE and *i*∈{*S*,*F*,*I*}, we also define Ω_*j*_ as the intersection between the observation footprint and the continent's grounding line, because height loss by all processes (except GIA) do not contribute to changes in the geoid beyond this line; see Zammit-Mangion *et al.* (2014) for details. We evaluate the integral for GRACE by fine gridding the footprint domain although Monte Carlo integration can also be used (Katzfuss & Cressie, 2011).

Rewriting 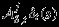 as a Wiener integral, it is straightforward to show that the observed fine-scale variation at the *j*^*th*^ observation is distributed as


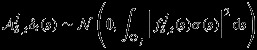
(5)

where *σ*(***s***) is the spatially varying variance of the uncorrelated process. For homogeneous fine-scale variation, when averaging over the footprint area, 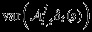 scales as 1/|Ω_*j*_| while when accumulating (as is the case with GRACE), as |Ω_*j*_|. The latter implies, at first sight, that this variation can have a large impact on the GRACE readings. However, placing realistic values for the fine-scale variation, which roughly correspond to the maximum *a posterior* estimates in Section 3.2, yielded a variation that was three orders of magnitude lower than that of the GRACE signal itself 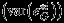. Because of this parameter insensitivity, we omitted the effect of fine-scale variation on the GRACE readings by setting 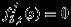 for these data. This assumption simplifies estimation of the fine-scale variation parameters that are, in any case, mostly informed through the altimetry data.

### 2.2. The process layer

The third layer describes the evolution of the multivariate spatio-temporal model. Three of the variates are spatio-temporal, while the fourth, GIA, is spatial (repeatedly observed at each time point). For all the three spatio-temporal processes, we assume a spatio-temporal auto-regressive process of the general form


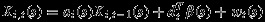
(6)

where *a*_*i*_(***s***) is a possibly spatially varying auto-regressive parameter, 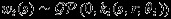, where 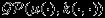 denotes a Gaussian process (Rasmussen & Williams, 2006) with mean *μ*(·) and kernel *k*(·,·), where *k* is not necessarily spatially invariant, 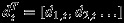 are covariates and ***β***(***s***) the respective spatial weightings, which need to be estimated. We now discuss the models for each of the spatio-temporal processes in turn.

#### 2.2.1. Ice dynamics/subshelf melting

Height changes due to ice dynamics, and subshelf melting is assumed to be relatively smooth over the short time scales considered, that is they follow a linear trend. This assumption follows from studies that show approximately linear temporal variation in ice discharge (Rignot *et al.*, 2011b). The ensuing model for this process is


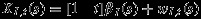
(7)

where 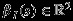 represents the spatially varying mean change and accelerating change in height, respectively, due to ice dynamics. We assume that both ***β***_*I*_(***s***) and the disturbance ***w***_*I*,*t*_(***s***) have small spatial scales and are assumed spatially uncorrelated following discretisation. Prior distributions for ***β***_*I*_(***s***) are given in Section 2.3. Fully specifying the statistics of *w*_*I*,*t*_ is crucial to avoid confounding with the fine-scale variation (which is mostly due to precipitation anomalies). We thus take 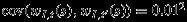 (i.e. we allow for confounding on scales of 1 cm year^−1^ if *t* = *t*′ and 0 otherwise. This choice also reflects the prior assumed (temporal) smoothness of height change due to ice dynamics and subshelf melting.

#### 2.2.2. Surface processes

Changes in height due to surface processes fluctuate rapidly at large (ice sheet) scales (Rignot *et al.*, 2011b). To check whether the same holds at small (20 km grid) scales, we fitted an auto-regressive(1) model to each spatial point from anomalies with respect to the 30-year mean for the output of the forward model RACMO. The auto-regressive coefficient was found to be positive in some places and negative in others. This variation, in addition to spatially varying amplitudes and length scales evident from RACMO, suggested that a heterogeneous model needs to be fitted to this process. As in Zammit-Mangion *et al.* (2014), we thus adopted the technique of fitting local homogeneous models on a coarsely gridded domain and then allowing the parameters to vary smoothly between the grid cells.

Locally, for each coarse-gridded cell, we assume that the surface process follows the local interactions model


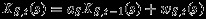
(8)

where *a*_*S*_ serves to define a random-walk space-time process (Stroud *et al.*, 2001; Zammit-Mangion *et al.*, 2012). Note that this is the auto-regressive interpretation of a space-time Gaussian field with separable covariance function *k*(*u*,Δ) = *k*_1_(*u*)*k*_2_(Δ) where *u*=∥***s*** − ***r***∥, Δ=|*t* − *t*′| and 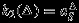. It can be shown (Storvik *et al.*, 2002) that in this case 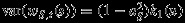.

For each cell, we let *k*_1_(·) (the spatial covariance function) be a Matérn function given by


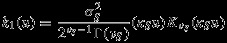
(9)

where 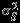 is the marginal variance, *κ*_*S*_ is the scaling parameter, *ν*_*S*_ is the smoothness parameter and 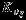 is the modified Bessel function of the second kind. Use of the Matérn is motivated by the dimensionality reduction methods employed in Section 2.4. The unknown parameters across all gridded cells are collected in 
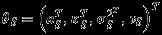
.

#### 2.2.3. Firn processes

Changes in height due to surface and firn processes are highly anticorrelated (more precipitation implies more compaction and *vice versa*). We capture this interaction by extending the spatial coregionalisation approach in Finley *et al.* (2007); Zammit-Mangion *et al.* (2014) to its spatio-temporal equivalent, termed the spatio-temporal linear model of coregionalisation by Choi *et al.* (2009). As in the spatial case, we model *X*_*F*,*t*_(***s***) as


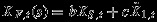
(10)

where 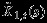 is independent but identically distributed as *X*_*S*,*t*_(***s***), that is


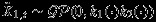
(11)

We can thus express the joint distribution as


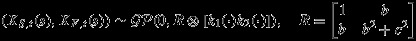
(12)

where ***R*** is a 2 × 2 correlation matrix composed of scalars *b* and *c* defining the point-wise interactions. Note that this definition of ***R*** is a scaled version of that in Zammit-Mangion *et al.* (2014) because here the marginal variance of the process is included in the definition of *k*_1_(·).

It can be shown, by comparing second moments, that the Gaussian field (12) is expressed in auto-regressive form as


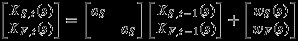
(13)

where


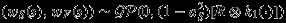
(14)

It is apparent here that coregionalisation does not induce off-diagonal elements in the propagation matrix. Interactions are induced solely through the correlated added disturbances. ***R*** is unknown and needs to be estimated.

#### 2.2.4. Glacio-isostatic adjustment

Glacio-isostatic adjustment is a slowly varying process with time scales on the order of thousands of years. For the 7-year period considered, we thus model it as a spatial process (which is repeatedly observed at each time point):


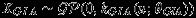
(15)

where *k*_*GIA*_ is a Matérn kernel, parameterised by some unknown parameter vector 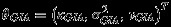.

#### 2.2.5. Fine-scale variation

**Figure 3:**
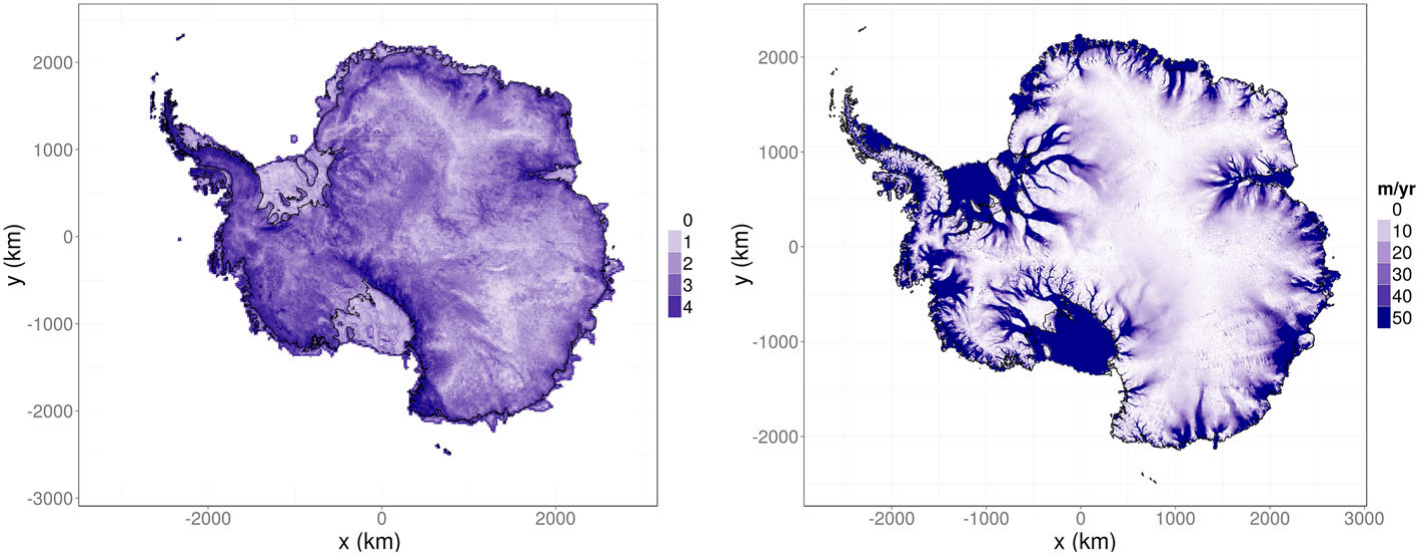
Left: Basis function used for small-scale variation, derived as described in main text. Right: Surface velocity map, used to shrink height change due to ice dynamics towards zero in areas of low velocity

We model the fine-scale variation *δ*_*t*_(***s***) as a stochastic volatility model as in Katzfuss & Cressie (2012)


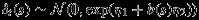
(16)

where *b*(***s***) is a basis function used to explain some of the fine-scale variation. Because we envision fine-scale variation to be present in high-relief regions, we use terrain roughness as a basis function *b*(***s***). The roughness function was derived from a high-pass channel image from the Moderate Resolution Imaging Spectroradiometer Mosaic of Antarctica Image map at 750 m resolution (Haran *et al.*, [Bibr b16]). The image was binned in 20 km boxes and the spread (standard deviation) of the image intensity in each box found. The logarithm of this spread incremented by 1 (to have a minimum roughness of 0), seen in Figure 3, left, was then used as the basis function for fine-scale variation. For identifiability reasons, we assumed that the statistics of fine-scale variation are temporally invariant. The parameters ***θ***_*δ*_=[*η*_1_,*η*_2_]^*T*^ are unknown and need to be estimated.

### 2.3. The parameter layer

Because of identifiability concerns, we adopt a hybrid approach to parameter estimation, in which some process parameters, **Θ**_1_=(***R***,***θ***_*GIA*_,***θ***_*S*_), are estimated from numerical models as in Calder *et al.* (2011), and the rest, **Θ**_2_=(***θ***_*δ*_,*γ*^2^,*θ*,***β***_*I*_(***s***)), are estimated from the data in an MCMC scheme. The estimation procedure for the former group is described in Section 3.1. For the latter, we apply the following prior beliefs:

- ***θ***_*δ*_=[*η*_1_,*η*_2_]^*T*^: These parameters describe the second-order statistics of the fine-scale variation and can take values on the real line. For this reason, we let
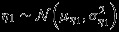
(17a)
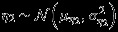
(17b)
  where the hyper-parameters 
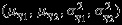
are set following a preliminary study employing a simplified process model and only one of the data sets (Section 3.2).
- *θ*: This parameter describes the extent of averaging within a neighbourhood of GRACE observations and should be positive. However, from (2), observe that ***P***_*G*_ is invariant to *θ* when (*#iθ*) exceeds 0.9, which occurs at *θ*≈0.14for the observation arrangement shown in Figure 2. For this reason, we constrain *θ* < 0.14 by equipping it with a uniform prior
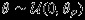
(19)
  where 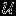 denotes the uniform distribution and *θ*_*p*_=0.14.
- *γ*_*G*_: This parameter allows for variance inflation of the GRACE signal. To obtain a tractable conditional distribution, we let
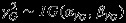
(20)
  where *IG* is the inverse-Gamma distribution and the hyper-parameters are configured such that the 95 percentile *q*_0.95_=400 and the 5 percentile *q*_0.05_=1, reflecting our belief that we do not expect the variance inflation to be outside the range [1,400]. Suitable values are 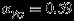
and 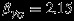.
- ***β***_*I*_(***s***): The spatially distributed parameters *β*_1_(***s***),*β*_2_(***s***)define the linear and accelerating change in height due to ice sheet processes, respectively. The marginal variances of these are set to be high for regions where the ice is flowing fast, and low (with a shrinkage to zero) otherwise, to reflect the fact that height change due to ice dynamics at very low speeds (<10 m s^−1^) is unlikely. We thus let

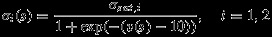
(21)

where we set *σ*_*sat*,1_=15 m year^−1^, *σ*_*sat*,2_=0.5 m year^−2^for one sector of Antarctica, the Amundsen Sea Embayment (which is known to exhibit large accelerations, see Scott *et al.*, 2009) and *σ*_*sat*,2_=0.1 m year^−2^elsewhere. Here, *v*(***s***)is the ice velocity obtained from synthetic aperture radar data (Rignot *et al.*, 2011a), supplemented with balance velocities (Bamber *et al.*, 2000) where data are missing. The reconstructed velocity map used is shown in Figure 3, right. Note that although we have a spatially continuous specification for these parameters, they will be estimated following discretisation (together with the processes) on a triangulation within a Bayesian setting, see Section 2.4.

### 2.4. Gaussian field approximation

**Figure 4:**
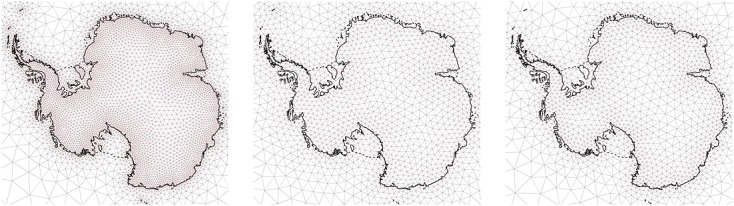
Meshes used to reconstruct the spatio-temporal processes. From left to right: (i) *X*_*I*,*t*_(*s*),*β*_*I*,1_(*s*),*β*_*I*,2_(*s*); (ii) *X*_*S*,*t*_(*s*),*X*_*F*,*t*_(*s*); and (iii) *X*_*GIA*_(*s*)

While convenient for modelling, Gaussian field spatio-temporal process models are not suitable for large-scale inference because the computational complexity of a required Bayesian update scales as *O*(*m*^3^) where *m* is the number of data points. The dependence on *m*^3^ is prohibitive in several applications, including the one considered here. One remedy is to project functions from the original, infinite-dimensional space 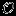 onto some lower-dimensional space 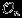 through a set of basis functions 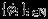. A natural way to approximate Gaussian fields is by noting that if these are of the Matérn type, they can be treated as solutions to stochastic partial differential equations (SPDEs) of the Laplace type (Lindgren *et al.*, 2011). It can be shown that the projection onto 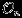 of such an SPDE with compactly supported basis functions yields a Gaussian Markov Random Field (GMRF) with favourable computational properties (Rue & Held, 2005). The basis functions we employ are ‘tent’ functions constructed on the triangulations shown in Figure 4.

The GMRFs are obtained by decomposing the spatio-temporal field at each time point and the spatially distributed parameters (*β*_1_(***s***),*β*_2_(***s***)) as a weighted sum of the tent basis functions, that is we let *X*_*i*,*t*_(***s***)≈***φ***_*i*_(***s***)^*T*^***x***_*i*,*t*_ and *β*_*I*,*i*_(***s***)≈***φ***_*i*_(***s***)^*T*^***β***_*I*,*i*_. This decomposition allows us to rewrite the second data layer as


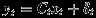
(22)

where 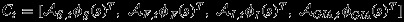, 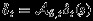 and the multivariate spatio-temporal model (ignoring GIA for now) is


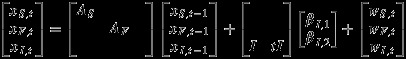
(23)

where ***I*** is the identity matrix of appropriate size. Here, ***A***_*S*_≡***A***_*F*_ describe the temporal propagation of the processes and are diagonal under the local separability condition assumed. ***β***_*I*,1_ and ***β***_*I*,2_ are discretised versions of *β*_*I*,1_(***s***) and *β*_*I*,2_(***s***), and 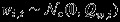 is added random forcing, where 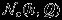 denotes the normal distribution with canonical parameters ***h*** and ***Q***. For the surface field, ***Q***_*w*,*S*_ (which describes heterogeneous fields) is obtained by assuming that ***w***_*S*,*t*_ is the discretised solution to an SPDE with spatially varying parameters, as discussed in detail in Zammit-Mangion *et al.* (2014) and Lindgren *et al.* (2011) Section 3.2. The approach has the neat property that, *locally*, ***w***_*S*,*t*_ can be interpreted as a discretised solution to a standard Matérn field. Details on how we obtain the spatially distributed parameters from RACMO are given in Appendix A.1. Following coregionalisation between the surface and firn fields, jointly we have that


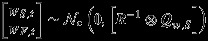
(24)

Finally, as discussed in 2.2.1, we set ***Q***_*w*,*I*_ diagonal, with precisions given by 1/0.01^2^.

We next combine the auto-regressive processes by defining 
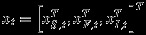
 and 
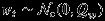
. The multivariate process can then be written in block form as


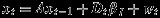
(25)

The full process describes Gaussian trajectories, so that we can represent the third layer as a GMRF over (***x***,***β***_*I*_) where 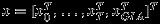. Let 
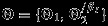
, that is the set of all unknown parameters except ***β***_*I*_. Then,


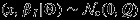
(26)

The full precision structure ***Q*** is obtained by considering the joint latent process, which decomposes as follows:


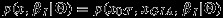
(26a)


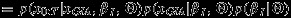
(26b)


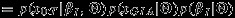
(26c)


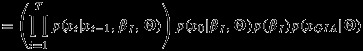
(26d)

where (26c) follows from (i) ***x***_0:*T*_ being conditionally independent of ***x***_*GIA*_ given (***β***_*I*_,**Θ**) and (ii) ***x***_*GIA*_ being conditionally independent of ***β***_*I*_ given **Θ**. Equation 26d follows from ***β***_*I*_ being independent of **Θ**. The prior 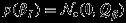 (where ***Q***_***β***_ follows directly from (20)), while the initial process distribution is defined as


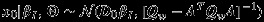
(31)

We choose the precision ***Q***_0_:=***Q***_*w*_−***A***^*T*^***Q***_*w*_***A***, and not that of the respective stationary distribution, as it is sparse for sparse ***Q***_*w*_ and diagonal ***A***, thus preserving computational tractability. Further, the implied covariance matrix is representative for diagonal ***A*** and ***Q***_*w*_; in particular, if ***A*** = *c**I*** for *c*∈(−1,1), the induced covariance is the same as that of the stationary distribution (see Hamilton, 1994, Section 10.2).

It can be shown that for this model the full precision matrix over (***x***,***β***_*I*_) is


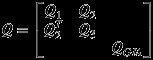
(32)

where ***Q***_1_ is the precision matrix without exogenous inputs (see Rue and Held, 2005, Ch. 1 for a univariate equivalent)


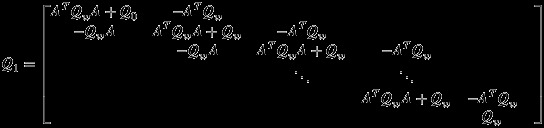
(33)

and ***Q***_2_,***Q***_3_ are defined as


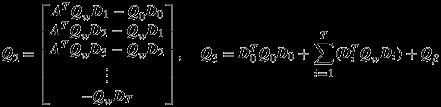
(34)

Because of the tractability of the linear state-space model, we have combined ***β***_*I*_ with ***x***, although the former are ‘parameters’ in the conventional sense. Because we have represented (***x***,***β***_*I*_) jointly, by concatenating across time we can rewrite the observation and process layers as follows:


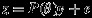
(31a)


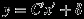
(31b)


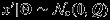
(31c)

where ***C***′ = [***C***,**0**], 
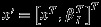
.

## 3. Parameter estimation and inference

### 3.1. Estimation of parameters from geophysical models

**Figure 5:**
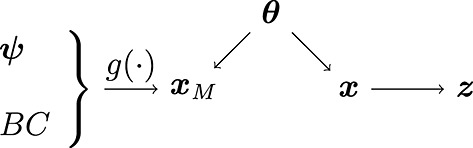
Assumed relationship between the geophysical (deterministic) model output *x*_*M*_=*g*(*ψ*,*BC*), the true process *x*, the observations *z* and underlying parameters *θ*. Conditioned on *θ*, *x*_*M*_ and *x* are assumed to be independent. The observations *z* are recordings of the true process *x*

Because we have three classes of data (altimetry, gravimetry and GPS) and four processes to infer, the problem is under-determined, rendering process-related parameter estimation from observations particularly difficult. We hence make use of numerical (geophysical) models for estimating the process parameters, namely **Θ**_1_. Specifically, denote the vector of physical observations as ***z*** and numerical model output as ***x***_*M*_ (we use the symbol *x* to emphasise that we treat numerical model outputs as *directly observed* data). ***x***_*M*_ is the output of a deterministic model *g*(·) run with suitably defined parameters ***ψ*** and boundary conditions *BC*. The first assumption we make is that the true (unobserved) process ***x*** and ***x***_*M*_=*g*(***ψ***,*BC*) are conditionally independent, which we model as *p*(***x***,***x***_*M*_|***θ***) = *p*(***x***|***θ***)*p*(***x***_*M*_|***θ***) for some parameter ***θ***∈**Θ**_1_, see Figure 5.

The second assumption is that ***x***_*M*_ is highly informative on ***θ*** so that we can safely restrict the posterior parameter density to a point estimate: 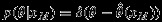. Then,


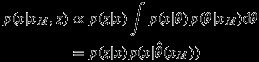
(32)

Equation (32) suggests a two-stage approach to inference. First, find the maximum likelihood estimate of ***θ*** under ***x***_*M*_: 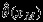. Second, carry out a Bayesian update on ***x*** using ***z*** and 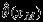 as plug-in values in *p*(***x***|***θ***). This approach is similar to that shown in Calder *et al.* (2011). Details for this first stage as applied to the present application are given, for the relevant processes, in Appendix A.

### 3.2. Markov chain Monte Carlo implementation

In order to carry out inference over ***x*** and **Θ**_2_, we need to complete the model specification by eliciting prior beliefs over ***θ***_*δ*_. We do this by using information from the log-likelihood of these parameters, evaluated at several points on a much simplified model. The log-likelihood reveals that that *η*_1_ and *η*_2_ are highly correlated because of the large flat surface in the continent's interior; we therefore also transform these parameters to aid mixing. Details for this step are given in Appendix B.

The sheer size of the problem under study devotes special attention. The combined dimensionality of (***x***,***β***_*I*_) is over 80 000, we have over 300 000 observations, crucially some of which have a large spatial support. The time taken to compute the Cholesky factor of ***Q*** following an approximate minimum degree permutation, and the marginal variance using the SuiteSparse package (Davis, 2011), required about 5 h on a mid-end server. This is prohibitive even for the vanilla implementation of the popular integrated nested Laplace approximations (INLA, Rue & Martino, 2009) package. We deal with this by implementing a scheme that can be parallelised and implemented on a distributed system.

We divide the state-space ***x***′ into *d* contiguous spatial blocks as in Zammit-Mangion *et al.* (2014). Denote the indices of the *ith* subset of ***x***′, ***x***^′*i*^, as 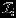 and let 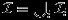. The conditional posterior distribution for the *i*^*th*^ block is given by 
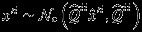
 where


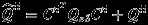
(33a)


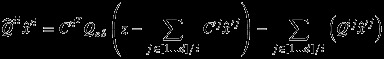
(33b)

where 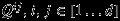 is the matrix composed of the rows in ***Q*** with indices in 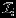 and the columns of ***Q*** with indices in 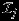 and where ***C***^′*i*^ is the matrix composed of the columns in ***C***′ with column indices in 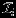. Here, 
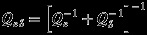
, ***Q***_*e*_=prec(***e***) and ***Q***_*δ*_=prec(***δ***).

**Figure 6:**
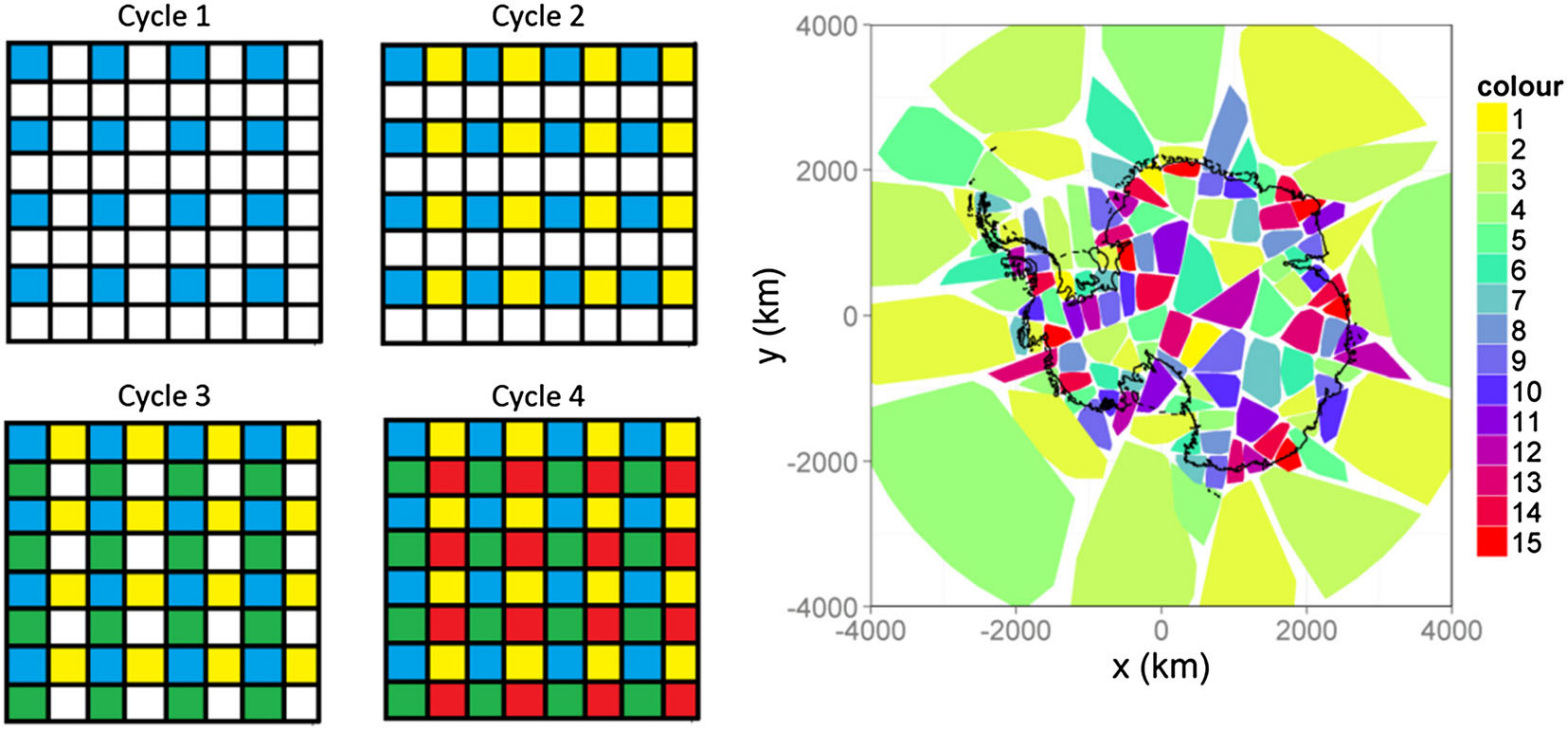
Left: Dispatching blocks under an 8 × 8 raster blocking scheme. A complete sample is obtained after the fourth cycle. Right: The final partitioning after applying the Kernighan–Lin algorithm seven times and a greedy colouring algorithm

In this work, we extend the approach by blocking with a view to parallelisation over a multinode architecture. Parallelisation is possible in Gibbs sampling if one can find groups of {***x***^′*i*^} that are independent when conditioned on the rest of the state space and the data. Groups because groups within this set, say 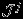, can then be updated in any order desired (or, indeed, even in parallel) for a given deterministic sweep strategy. As an example, consider the following scenario, depicted in Figure 6, left. Here, we have divided the entire state space into a raster of 8 × 8 blocks. Assume each block is conditionally independent of every other block given the neighbouring blocks and the data, then we can obtain a sample of ***x***′ by sampling all nonadjacent blocks in parallel. For this simple case, we only need four cycles, one for each block set 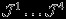; a potential speed-up of 64/4 = 16 is achievable. More generally, a simple parallel Gibbs sampling proceeds as follows: first, instruct computational nodes to sample from blocks with index in 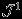 in parallel, wait for the cycle to complete, update and distribute the latest sample and repeat for 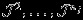. This sampling strategy is known as chromatic sampling (Gonzalez *et al.*, 2011), because the assigning of conditional independent groups is akin to the problem of graph colouring (Molloy & Reed, 2002): the problem of finding a colour arrangement such that no two neighbours in a graph have the same colour.

We cannot employ a simple raster blocking method and a four-colouring scheme (which is possible for all graphs on a plane, see Gonthier, 2008) for two reasons. First, the key drawback with raster partitioning is the unequal load distribution when employing variable mesh density (as in this problem). Blocks of equal size will, in general, contain drastically different numbers of vertices. This, in turn, will cause computer nodes in a high-performance environment to ‘wait’ unnecessarily for the block containing the largest number of vertices to be completed before the next cycle can begin. We remedy this problem by dividing the graph into subgraphs of roughly equal size using the *Kernighan–Lin* algorithm (Kernighan & Lin, 1970). In this study, we use the implementation by Ladroue ([Bibr b25]) seven times in order to subdivide the graph into 128 roughly equal-sized partitions. The final partitioning scheme we used in our problem is shown in Figure 6 (right): with this blocking strategy, we obtained a median block size of 609 vertices with a minimum of 590 and a maximum of 750, which is indicative of a good load balance.

The second problem is that, *conditioned on the data*, the adjacency graph over the blocks is no longer a sufficient constraint for conditional independence. This is made apparent from the second term within the brackets of (33b). This term implies that because of dense rows in ***C***′, 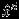 may even depend on states that are in blocks not immediately adjacent to block *i*. Thus, when colouring the graph, in addition to the constraint that no two adjacent blocks have the same colour, we have to apply the constraint that no observation can be informative of more than one block of a given colour. This is particularly relevant to the present problem because GRACE has a support that spans multiple blocks. We coloured the graph using a greedy algorithm on a breadth-first-search ordering of the vertices. The final arrangement with the lowest amount of colours, 15 in this case, is shown in Figure 6, right.

We grouped the GIA field on its own (i.e. assigned it a colour of its own) because it is highly spatially correlated. With 1832 vertices, the GIA block is about three times larger than any other block so that the theoretical maximum speed-up is thus 129/(15 + 3) = 7.2. In practise, we saw a fivefold speed-up over use of a single thread when sampling the state space only, which is remarkably close given the communication overhead, notable in this case because of message passing of the updated samples. Sampling of all the other parameters (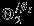, see Section 3.3) needs to be done sequentially; the final speed-up we obtained for one whole MCMC cycle was of about three; however, this is still significant given the time needed to generate one sample. Moreover, this approach extends nicely to larger state spaces, if required. We generated 16 000 samples on a high-performance computer (having 16 2.6 GHz SandyBridge cores per node and 4 GB memory per core) using four nodes and 24 parallel threads with RMpi in 42 h.

### 3.3. Sampling the parameters and diagnostics

Following an updated sample of ***x*** and ***β***_*I*_, we sample from the conditionals of the remaining parameters, defined as follows


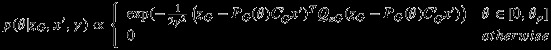
(34a)


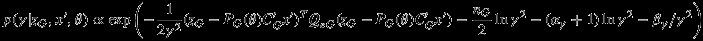
(34b)


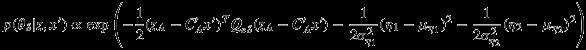
(34c)

where *n*_*G*_ is the number of GRACE observations, ***Q***_*eG*_=prec(***e***^∗^) (recall that ***e***^∗^ is the error prior to inflation), 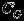 is the submatrix of ***C***′ corresponding to the GRACE observations and 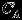 is that corresponding to the altimetry (ICESat and EnviSat). Note that (34c) is independent of *θ* and *γ* as a result of the assumption 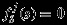 for the GRACE observations in (5).

From this note that only *γ*^2^ can be sampled from directly. For *θ* and ***θ***_*δ*_, we initially carried out a Metropolis-within-Gibbs step by proposing from a Gaussian distribution centred at zero with variance 0.1^2^. However, the resulting effective sample size from this scheme was remarkably low, prompting us to use univariate slice sampling instead (Neal, 2003), which considerably decreased autocorrelation. We implemented the stepping-out methods and adapted the interval width used for the first 50 samples, following which the interval was fixed. These samples were then discarded.

Visual inspection of the traceplots confirm good mixing of the chains. Geweke diagnostics and autocorrelation plots on these traces also support convergence. To assess convergence in ***x*** and ***β***_*I*_, we took 2000 variables at random from ***x***′ and carried out Geweke diagnostics on each of the traces. The z scores from the diagnostics were then verified to be normally distributed (expected under the assumption of trace independence, a reasonable one given the continent's size and correlation lengths). Geweke diagnostics were carried out using the R package coda.

## 4. Validation, visualisation and results

In this section, we summarise the main inferential results. In Section 4.1, we test the model using an independent data set and use the results to expose some limitations of the approach, while in Section 4.2, we focus on the marginal fields. Finally, in Section 4.3, we concentrate on mass balance estimates, that is we determine where and by how much Antarctica is losing or gaining mass. In all Antarctica plots presenting our results, we use bilinear interpolation to reconstruct the mesh surface, overlay the triangulation to convey the native process resolution and stipple with green dots where the absolute posterior mean is greater than one posterior standard deviation.

### 4.1. Diagnostic check using a separate remote sensing data set with pivoted Cholesky residuals

**Figure 7:**
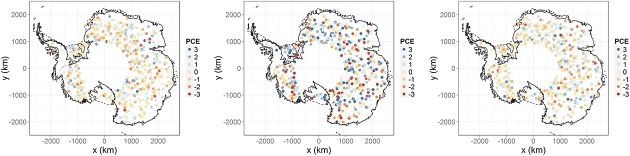
Pivoted Cholesky residuals (PCE) of validation data using real data (centre) and pseudo-data generated from the posterior distribution (left and right)

In this section, we check the model using data from an altimeter on EnviSat's predecessor, the European Remote-sensing satellite-2 (ERS-2). Because of process smoothness, we compute residuals that are ‘corrected’ for correlation through the pivoted Cholesky residuals, which are derived from the covariance matrix of the residuals (Bastos & O'Hagan, 2009). The ‘pivot’ in the pivoted Cholesky decomposition of the matrix sorts the residuals as follows: the first element is that with the highest variance, the second element is the largest variance conditioned on the first element and so on. In a spatio-temporal setting, locations in the vicinity of each other should be far apart in the ordering, even if they have similar marginal variances, that is the variance of the first point in a close pair should be low when conditioned on the second point if correlations are correctly captured. The advantage of this approach is that proximity and spatio-temporal correlation are taken into account when visualising the residuals. As such little correlation should be visually apparent when inspecting plots of the pivoted Cholesky residuals (as opposed to when visualising the standardised residuals). We carry out this diagnostic check by choosing 500 validation points from the ERS-2 data. The pivoted Cholesky residuals are seen in the centre panel of Figure 7 where only some correlation is apparent, indicating that the chosen spatial structure of the model is not implausible.

A pivoted Cholesky residual plot can be put into perspective by carrying out a ‘Turing test’. This involves sampling validation data points (at the space-time locations) from the predictive distribution, re-evaluating the pivoted Cholesky residuals for this pseudo data and comparing the resulting plots to that of the true data. This informal test checks whether we can spot the real-data case from the pseudo-data case. Overall, from Figure 7, we find it relatively easy to pick out the pseudo-data cases from the real-world case, the latter exhibiting more extreme residuals, representative of overconfidence. A density plot of the pivoted Cholesky residuals (not shown) indicates that the residuals are centred at zero but also confirms an overall overconfidence. It is not clear whether this is a result of model mis-specification or an issue with the residuals in the ERS-2 data set; however, it does hint at the possibility of our posterior estimates being slightly overconfident.

### 4.2. Posterior fields

**Figure 8:**
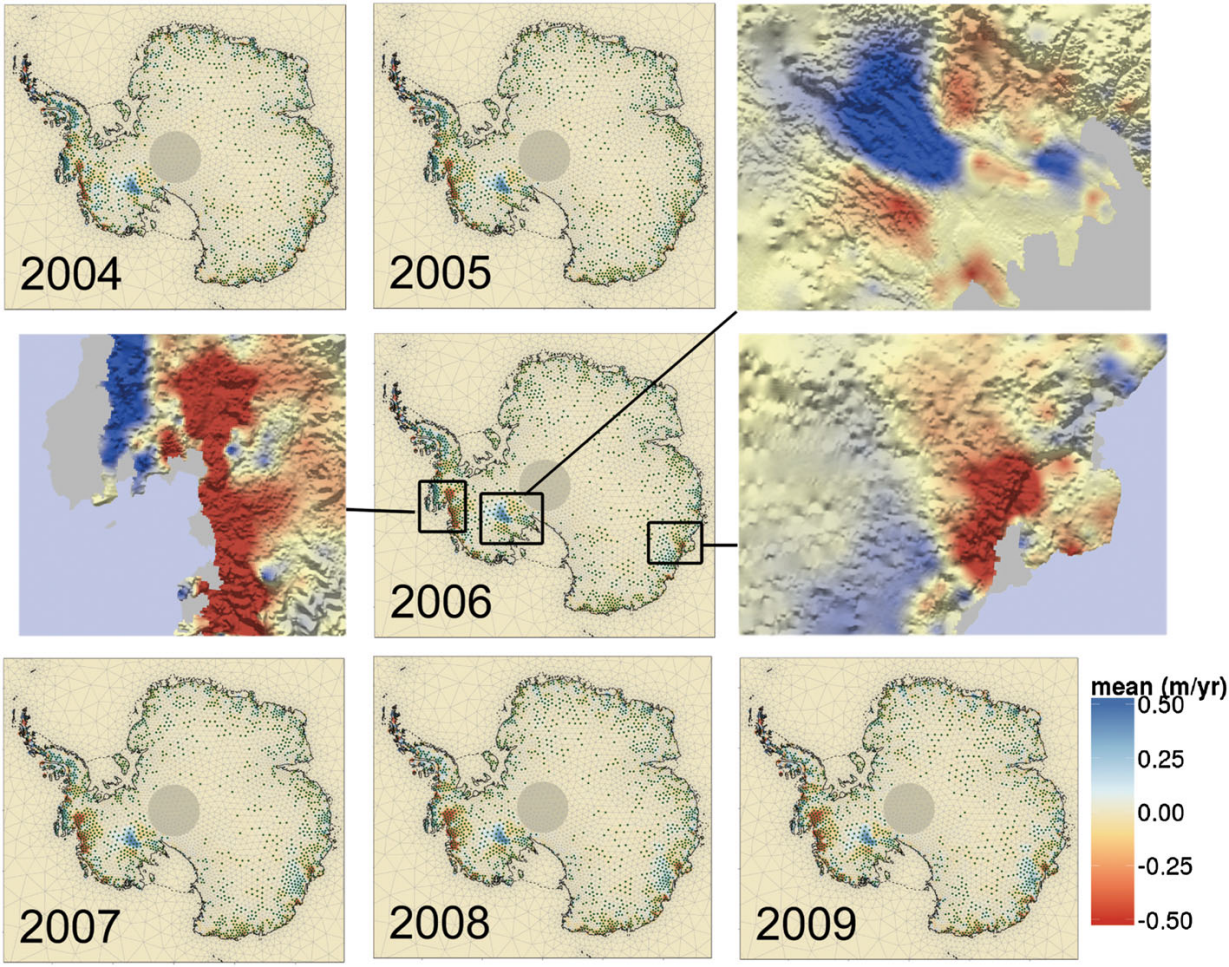
Evolution of height rates due to changes in ice dynamics from 2004 to 2009. Insets show close ups of the Pine Island Glacier (centre left), the Kamb Ice Stream (top right) and the Totten Glacier (centre right) in 2006, overlayed over the bedrock topography extracted from Bedmap2 (Fretwell *et al.*, 2013). Height changes over ice shelves (enclosed between the dashed and solid lines and grayed out in the insets) are omitted as these do not contribute to sea-level change

**Figure 9:**
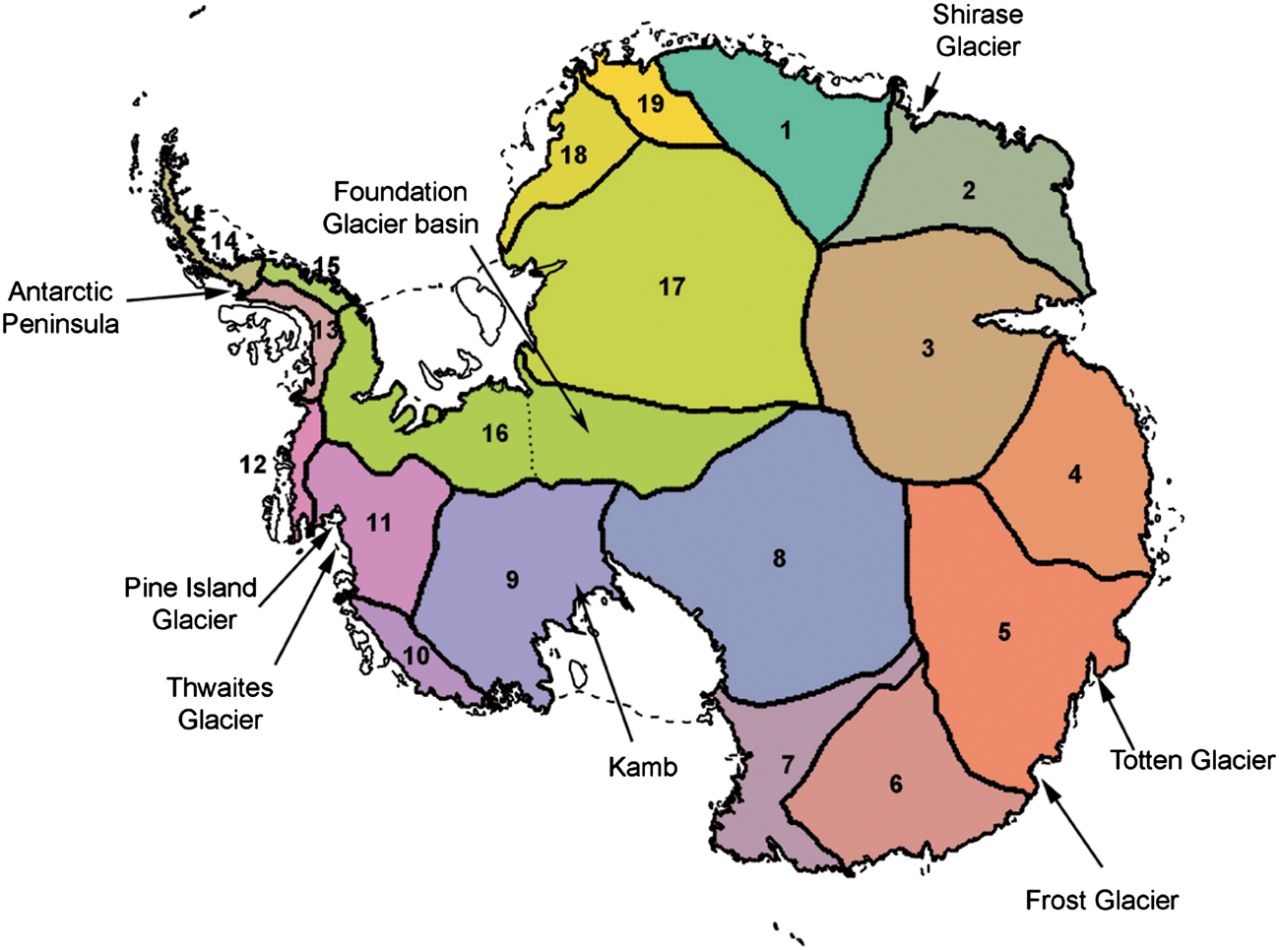
Basin locations corresponding to Rignot *et al.* (2011a) and landmarks. The West Antarctic ice sheet is composed of Basins 9, 10, 11, 12 and part of 16, the East Antarctic ice sheet of Basins 1–8,17–19 and part of 16 and the Antarctic Peninsula of Basins 13–15. The Foundation Glacier basin (east part of Basin 16) is associated with the East Antarctic ice sheet (Section 4.3)

In Figure 8, we show results for the mass rates due to ice dynamics for the 7 years under consideration together with uncertainty estimates. These results corroborate the spatial results of Zammit-Mangion *et al.* (2014) where only West Antarctica was considered. The most prominent features include the negative, accelerating rates in the Pine Island Glacier and Thwaites Glacier (inset) and an overall ice build-up (with no significant acceleration) in the Kamb area (inset). In East Antarctica, several expected features are also apparent, most notably the considerable mass loss occurring in the Totten Glacier (Velicogna & Wahr, 2013, inset) and areas surrounding the Frost glacier (Rignot, 2006), both of which are grounded below sea level and are hence considered potentially unstable (for basin definitions and landmarks, see Figure 9). Some glaciers, such as Shirase glacier towards the top of the plot (reported to be largely in equilibrium by (Pattyn & Derauw, 2002)), are shown to be thickening. We note that glacier thickening at large rates is physically improbable (except in the Kamb area, Ng & Conway, 2004) and that here there could be confounding between changes due to ice dynamics and persistent positive local precipitation anomalies. We omit changing height thickness over the ice shelves in the plots, as this is not a contributor to SLR.

**Figure 10:**
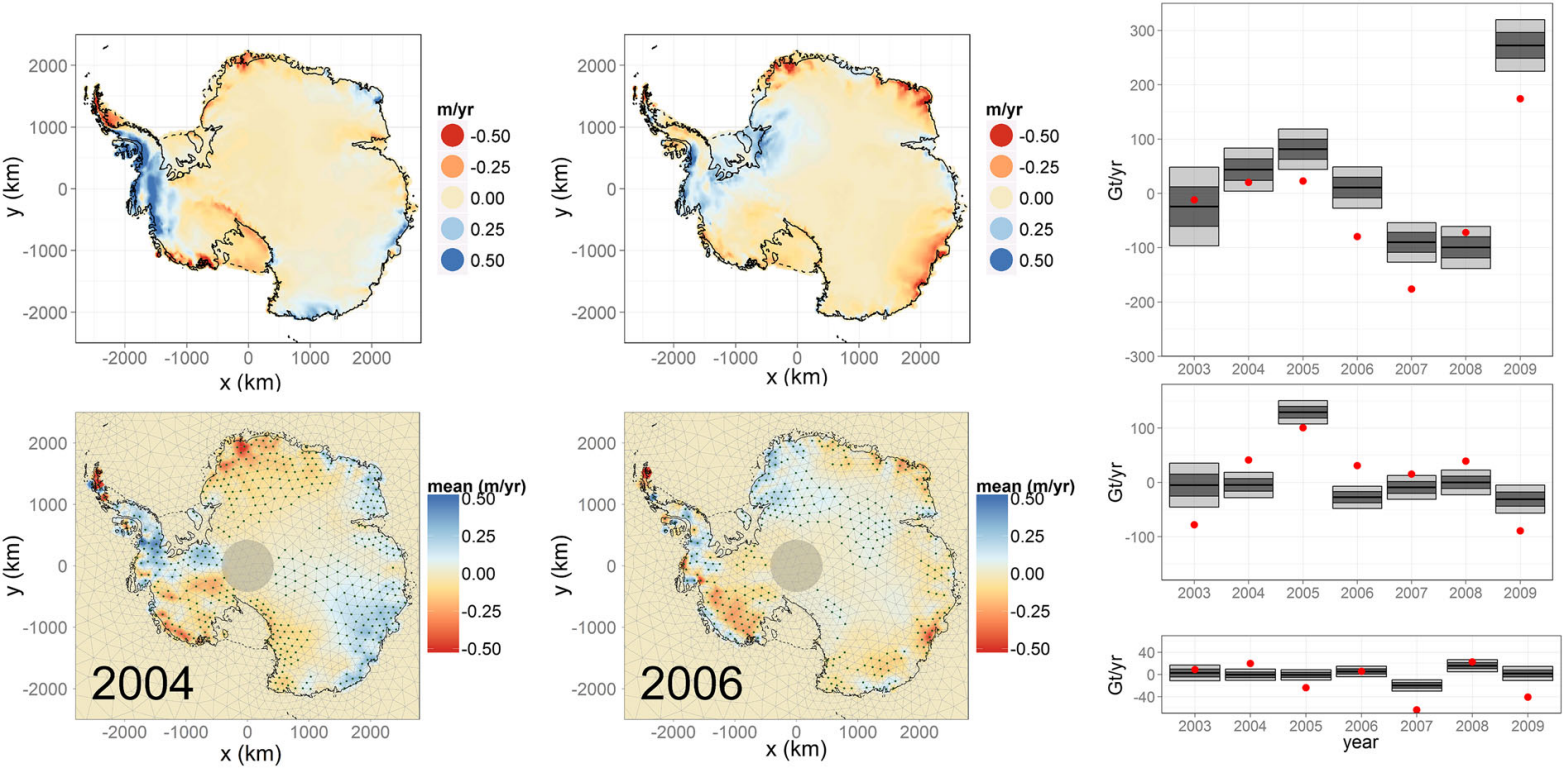
Left: Anomalies from a 30-year mean in the output of regional climate model (top) compared to the estimated surface process height rates (bottom) from the framework for 2004 and 2006. Right: Estimated rate of mass change due to surface processes (black bars) and the values given by the regional climate model (red dots) for the East Antarctic ice sheet (top), West Antarctic ice sheet (centre) and Antarctic Peninsula (bottom). The 1*σ* and 2*σ* levels are denoted by the dark and light shadings, respectively

In Figure 10, we provide a comparison of posterior estimates for height changes due to surface processes and those provided by the regional climate model, RACMO. When considering aggregates (right), the positive-negative trend is clearly followed by RACMO although, for East Antarctica, a bias is evident, possibly because of a systematic underestimation of the snowfall anomaly by RACMO for 2004–2007 (van Wessem *et al.*, 2014). It should be noted that East Antarctica is one of the most poorly sampled places on the planet in terms of meteorological observations and therefore also relatively poorly constrained in RACMO. Although in most cases outside our 95% credibility intervals, we find it encouraging that the shape and phase of the anomaly patterns could be reconstructed. Essentially, this result shows that RACMO reconstructs the temporally high frequency, spatially mid-frequency signals evident in satellite altimetry data but with differences in the amplitude of the signal.

**Figure 11:**
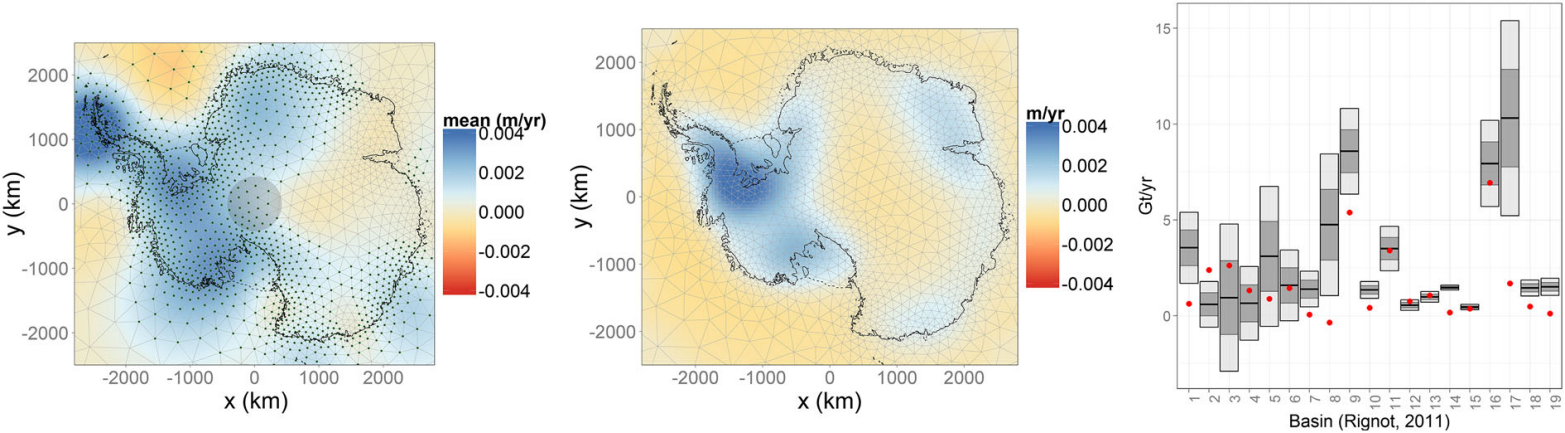
Left: Posterior estimate of *x*_*GIA*_. Stipples mark the states where |*μ*|>1*σ*. Centre: The glacio-isostatic adjustment (GIA) model output of Ivins *et al.* (2013), IJ05R2. Right: The estimated total change of mass due to GIA on a basin scale (black bars) and the value given by IJ05R2 (red dots). The 1*σ* and 2*σ* levels are denoted by the dark and light shadings, respectively

In Figure 11, we provide our reproduction for the GIA field and compare it to a forward numerical model, IJ05R2, which we used to set the parameters on ***x***_*GIA*_. Notably, as in IJ05R2, we observe a positive trend (uplift) of 2–4 mm year^−1^ GIA over West Antarctica and an overall negative signal (subsidence) towards the pole and over much of East Antarctica. The main discrepancy apparent in Figure 11 (right) is the large positive rate observed in our results in basins 1, 17–19, which correspond to the upper part (in the figure) of the East Antarctic ice sheet. We caution that GPS readings used here have not been corrected for the elastic response of the lithosphere and that we are using release 1 of the GRACE mass-concentration data set (work on a second release is underway). Consequently, the GIA results (and, to a lesser extent, the SLR contribution estimates of Section 4.3) are not definitive and presented as illustrative of the framework capability.

### 4.3. Estimates of sea-level rise contribution

Multivariate spatio-temporal modelling allows us to discriminate between the processes, in order to obtain mass-balance estimates from assumed density profiles, the same profiles that are used to translate the observed height changes to the mass changes observed by GRACE. As discussed in Zammit-Mangion *et al.* (2014), several authors have provided SLR contribution estimates for Antarctica. Here, we compare to the GRACE-based method of Luthcke *et al.* (2013) (where the IJ05R2 model is subtracted from the GRACE signal) and the so-called input–output (IO) method, where mass balance is found by deducting the rate of ice discharge (obtained through satellite readings of horizontal ice velocity) from the surface mass balance, obtained from a regional climate model, in this case RACMO. We obtained these latter results from Shepherd *et al.* (2012), which are similar to those of Rignot *et al.* (2011a) but updated to include more recent data, particulary for measured ice thickness. We note that the mass imbalance is not strictly equivalent to SLR contribution when accounting for grounding line migration (Rignot *et al.*, 2011a), and thus, we can expect results from the IO method to be slightly more negative (although it is unclear whether the IO results had been corrected for this or not). To use similar basin definitions as these studies, we have allocated the Foundation Glacier basin to the East Antarctic ice sheet, see Figure 9.

**Figure 12:**
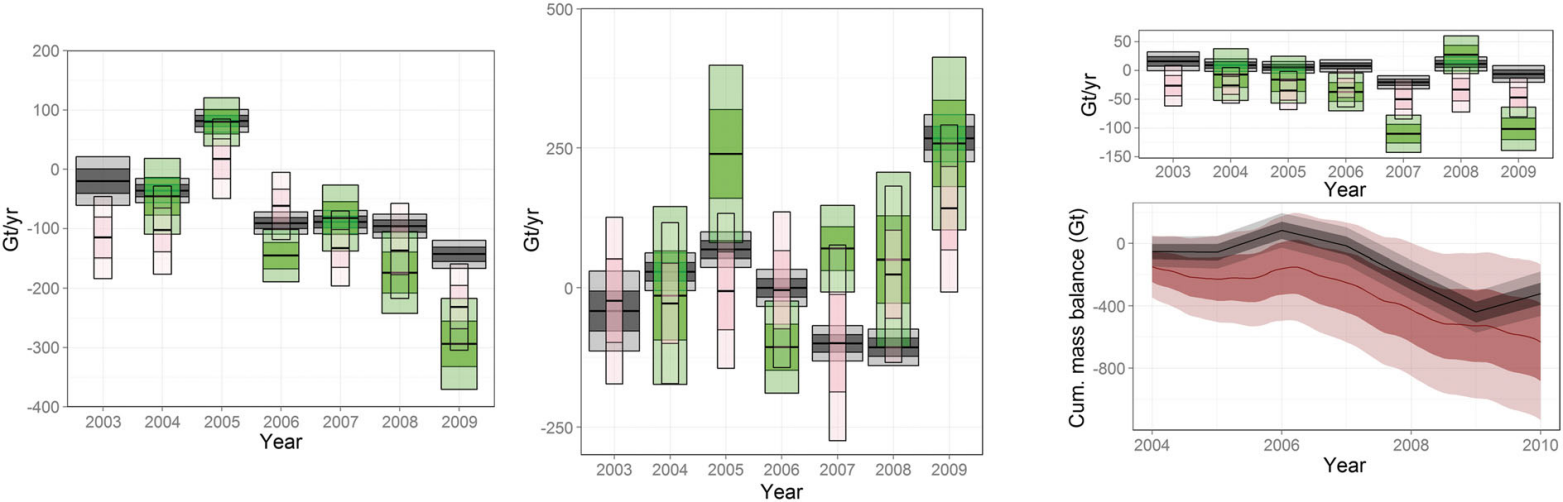
Mass-balance estimates (black) compared to those from the Gravity Recovery and Climate Experiment-based method of Luthcke *et al.* (2013) (green) and the IO method (pink) as reported in Shepherd *et al.* (2012). The 1*σ* and 2*σ* levels are denoted by the dark and light shadings, respectively. Left: West Antarctic ice sheet. Centre: East Antarctic ice sheet. Top right: Antarctic Peninsula. Bottom right: Estimated cumulative mass change throughout the period of study (black) compared to the estimate obtained when averaging over multiple methods (Shepherd *et al.*, 2012).

Estimates of SLR contribution for the West, East Antarctic ice sheets and the Antarctic Peninsula are given in Figure 12. Again, there is a broad agreement between the results. In the West Antarctic ice sheet, the positive anomaly in 2005 is clear for the three methods, as is the apparent negative trend in the latter years of the study. For the East Antarctic ice sheet, the conclusions are similar, in that the overall positive-negative pattern along the years is preserved, despite our solutions being temporally more smooth, probably because of the imposed auto-regression coefficients on the surface process prior which are mostly positive. The pattern we observe in the Antarctic Peninsula is reproduced in the other solutions; however, the large mass loss indicated in the other methods is not within our credibility intervals. This could be due to an optimistic prior belief in the definition over ***β***_*I*_ or a result of inaccurate representation of the field by the chosen mesh. It could also be due to overestimation of mass loss in the other methods for a region that is particularly challenging for both GRACE and the IO method because of the small scale at which ice dynamic takes place.

Finally, we compare the cumulative mass change obtained using our method with Shepherd *et al.* (2012), which is an unweighted mean estimate (using a meta-analysis) from three approaches (GRACE-based methods, IO-methods and altimetry + RACMO methods) and multiple data preprocessing methods. Our cumulative mass change follows the unweighted mean estimate but is less negative. We note, however, that a more recent version of the RACMO model, v2.3, produces a more positive snowfall rate by over 100 Gt year^−1^ compared with the version used in Shepherd *et al.* (2012) (van Wessem *et al.*, 2014). This difference would increase the integrated IO method results by the same amount, resulting in a lower mass loss rate for the unweighted mean in Shepherd *et al.* (2012). Our estimated SLR contribution of Antarctica during this period (2003–2009) is of 0.13 ± 0.08 mm year^−1^. This lies, as expected, between the the ICESat-based estimate of −0.06 ± 0.23 mm year^−1^ and the GRACE-based estimate of 0.16 ± 0.14 mm year^−1^ as reported in Shepherd *et al.* (2012) between October 2003 and December 2008; however, it is considerably less than the GRACE-based method of Luthcke *et al.* (2013), which reported 0.23 ± 0.07 mm year^−1^ for 2003–2010. The discrepancy with the latter is possibly due to the inclusion of 2010, a year in which the West Antarctic ice sheet has continued to witness increased ice loss (McMillan *et al.*, 2014).

## 5. Conclusion

In this paper, we have shown that spatio-temporal modelling is a powerful tool in assessing Antarctica's mass balance and contribution to SLR. Unlike other methods, its use of forward numerical models is up to the assumption of conditional independence (used only to estimate parameters) and is thus less restrictive and bias-prone than standard data assimilation procedures. Overall, we find that our results conform quite well with those making explicit use of numerical models, indicating that this approach may be used in the future to validate the models with remote sensing data. This is particularly useful in Antarctica where *in situ* data are difficult and sometimes impossible to obtain.

There are several ways in which the framework can be improved. (i) More work needs to be done on mesh generation, with special care taken in high-relief and narrow areas such as the North Antarctic Peninsula. A coarse mesh, in these regions, constitutes an inaccurate model representation, which may be the cause of the optimistic results obtained in this region. (ii) A comparison with the auxiliary data set from ERS-2 revealed that the fields are unbiased and that the modelled spatial correlation is suitable. However, an overall overconfidence was observed, which might be the cause of the relatively narrow credibility intervals seen in Section 4.2. This could be a result of model mis-specification, but it is also possible that the supplied errors with the preprocessed data are, in fact, optimistic. (iii) Process parameters are treated as fixed and assumed to be fully informed by geophysical models. In an MCMC scheme, parameter uncertainty can be easily included, although this would require the provision of posterior densities over the unknown parameters. To remedy this, one could still assume conditional independence but not restrict the posterior parameter density to a point estimate as we do here. (iv) The assumption of imposed (local) spatio-temporal stationarity might be a fair assumption for some of the processes considered here; however, this does not hold for ice dynamics, which is directional and exhibits spatio-temporal interactions (Hurkmans *et al.*, 2012). It would be possible within the current framework to consider a simplified transport model for this process (e.g. Wikle *et al.*, 2001) although this will come at some computational expense.

The key novelties of this work lie in the level of complexity and sheer size of the spatio-temporal model employed, the way in which numerical models were incorporated with large-scale multivariate spatio-temporal systems and the tools developed for inference over multiple-process architectures. Because the work is multifaceted, we have implemented an R package MVST that facilitates most operations described in this paper and provides a simplified analysis of that described here as a vignette. One notable useful feature of this package is the ease with which multiple observations, with spatially varying observation operator, can be considered with various types of multivariate spatio-temporal systems, including those decomposed on triangulations such as those considered here. It is envisioned that this package can be used to help set-up latent Gaussian multivariate spatio-temporal models in a variety of settings. Once the Gaussian model is set-up, it can be passed on for inference with other packages, such as INLA, although we also provide code for the elementary Gaussian case.

## Figures and Tables

**Figure A.1.**
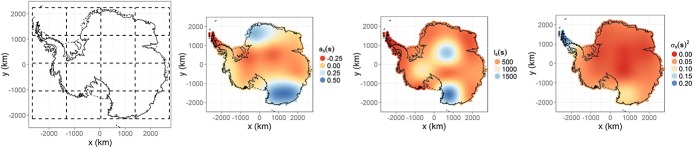
Heterogeneity in surface process obtained by fitting local spatio-temporal separable models and interpolating using Gaussian radial basis functions. From left to right: (i) Gridding up of the domain in blocks, the data in which are assumed to be independent. Separate models are fitted within each of these blocks individually; (ii) propagation coefficient *a*_*S*_(*s*); (iii) spatial range 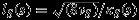 where *ν* = 1; and (iv) marginal variance 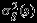

**Figure B.1.**
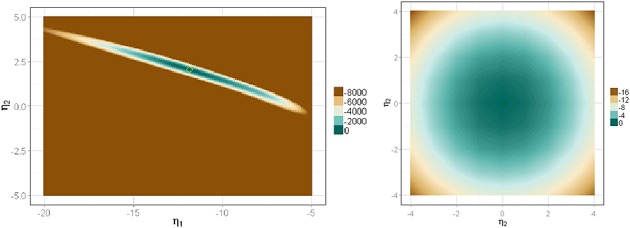
Unnormalised log-likelihood of fine-scale variation parameters *θ*_*δ*_ shifted so that the maximum is zero. Left: Pretransformation, with the diamond denoting the maximum likelihood. Right: Post-transformation

## Appendix A. Parameter estimation from numerical models

### A.1. Surface process parameters

For the surface layer, we estimated the Matérn and auto-regressive parameters from RACMO. In Zammit-Mangion *et al*. (2014), we provided estimates in the *spatial* setting by gridding the spatial domain and fitting homogeneous Matérn models to each block. Here, we adopt the same approach but instead fit a Gaussian separable *spatio-temporal* model to each block, with covariance function *k*(*u*,Δ) = *k*_1_(*u*)*k*_2_(Δ). We maximise the likelihood using a grid-based method for each block (for *ν*={1,2}) with 100 locations selected at random in each block. This provided estimates for  and *ν*_*S*_. For all blocks,  was estimated. The SPDE approach applied in Section 2.4 allows for smoothly varying parameters, whereby the local interpretation of the second-order properties are preserved. Therefore, in order to avoid discontinuities at the boundary, we subsequently obtained spatial maps for *a*_*S*_(***s***),*κ*_*S*_(***s***) and  by interpolating the points using Gaussian radial basis functions with standard deviations of 600 km. The blocking strategy and the resulting maps for all spatially distributed parameters are shown in Figure A.1.

As seen in Figure A.1, the temporal scales vary considerably. We observe an overall positive dependence in the lower right (in the plotted sense) and upper parts of the continent while an overall negative temporal dependence in the central band. The spatial scales are equally irregular and conform with those observed in Zammit-Mangion *et al*. (2014). Scales range from around 80 km at the peninsula (top left corner) to over 1500 km in the centre of the continent where precipitation is at a minimum. The marginal standard deviation further shows the spatially varying nature of height change due to surface processes and is as low as 2 cm year^−1^ in the centre of the continent, 20 cm year^−1^ on the coast and up to 40 cm year^−1^ at the wetter peninsula. A.2. Glacio-isostatic adjustment process parameters The GIA model we use to obtain ***x***_*M*_ is that of Ivins *et al*. (2013), IJ05R2. We carry out a simple grid search on the likelihood function for *ν* = 1 and *ν* = 2, which is maximised for .  corresponds to a range of approximately 1700 km, lower than that reported in Zammit-Mangion *et al*. (2014) (3000 km), which considered an earlier version of the model (Ivins and James, 2005).  mm is also lower than the previous estimate of 4.2 mm and reflects the lower GIA rates in the revised model. Importantly though, the overall expected magnitude (mm) and length scales (>1000 km) are of the same order. This implies that temporally invariant, low power, spatially low frequency components of the data will be attributed predominantly to the GIA process. A.3. Surface-firn interaction parameters. Because we are assuming a coregionalisation model, an estimate for***R*** is obtained by finding the empirical covariance matrix between the surface and firn processes from RACMO and a firn densification model (Ligtenberg *et al*., 2011) on a common grid. Here, we use the same estimate as that employed in Zammit-Mangion *et al*. (2014) given by

(A.1)

## Appendix B. Fine-scale variation hyper-parameters

For a GMRF with fine-scale variation, the likelihood of ***θ***_*δ*_ can be found analytically by integrating out ***x*** and ***β***_*I*_. Rewriting the observation layer as ***z*** = ***C***^′^***x***^′^+***δ*** + ***e***, the log-likelihood is given by

(B.1)

where ***Q***_*δ*_=diag(exp(−*η*_1_−***b****η*_2_)), ***b*** is the roughness *b*(***s***) evaluated at the observation locations, , which is also diagonal,

(B.2)

and *c*_1_ is a constant independent of ***θ***_*δ*_. Evaluating (B.1) for fixed *θ* and *γ* is very costly as the large footprint of GRACE serves to correlate all state variables *a posteriori*, resulting in dense matrices. For example, the Cholesky factor of , following fill-in reduction permutations, is over 5 GB when including GRACE observations, when compared to a few hundred Mb when using the point-referenced observations. However, we are solely interested in (B.1) to explore the parameter space and elicit a prior distribution over ***θ***_*δ*_. For this purpose, it is sufficient to consider the following simplified model:

That is, we consider only what ICESat (which has more coverage than EnviSat over rough terrain) is reading, assuming that all height change observed is due to ice dynamics or subshelf melting. We expect that the likelihood surface obtained in this way reflects an overestimation of ***θ***_*δ*_, because the other spatio-temporal processes (omitted in this simplified model) do explain some of the apparent variation. Using a simplified model allows us to explore the likelihood surface in considerable detail prior to applying a full MCMC scheme. The surface provides insights into how to transform the parameters for efficient sampling.

A plot of ln*p*(***z***_*I*_|***θ***_*δ*_) against gridded values for *η*_1_,*η*_2_ is shown in Figure B.1(left). The two parameters are, as expected, highly correlated because a large portion of the continent's surface is flat: the difference between a topographic-induced variation and a baseline variation is not pronounced. The surface plot, however, provides substantive evidence that the topographic-induced error is indeed the dominant component. We configured our prior based on this surface: we set  and , while for our uncertainty, because of use of the simplified model, we set . We next transform the fine-scale parameters. Using the gridded maximum as a starting value, we carry out a gradient-free optimisation to find the maximum likelihood and then compute the Hessian numerically (using the R package numDeriv). We use the Hessian and the mode to transform the variables into  such that (*η*1′,*η*2′) are approximately zero-centred uncorrelated Gaussian random variables. A plot of the likelihood surface for (*η*1′,*η*2′) is shown in Figure B.1 (right). In the MCMC scheme discussed, we sample directly from (*η*1′,*η*2′).
